# Tetra­kis[μ-4-(diethyl­amino)­benzoato-κ^2^ *O*:*O*′]bis­[(*N*,*N*-diethyl­nicotinamide-κ*N* ^1^)cobalt(II)]

**DOI:** 10.1107/S160053681005004X

**Published:** 2010-12-04

**Authors:** Tuncer Hökelek, Ertuğrul Gazi Sağlam, Barış Tercan, Özgür Aybirdi, Hacali Necefoğlu

**Affiliations:** a Department of Physics, Hacettepe University, 06800 Beytepe, Ankara, Turkey; b Department of Chemistry, Ankara University, 06100 Tandoğan, Ankara, Turkey; c Department of Physics, Karabük University, 78050 Karabük, Turkey; d Department of Chemistry, Kafkas University, 36100 Kars, Turkey

## Abstract

In the centrosymmetric binuclear title complex, [Co_2_(C_11_H_14_NO_2_)_4_(C_10_H_14_N_2_O)_2_], the two Co^II^ cations [Co⋯Co = 2.6199 (5) Å] are bridged by four 4-(diethyl­amino)­benzoate (DEAB) anions. The four nearest O atoms around each Co^II^ ion form a distorted square-planar arrangement, the distorted square-pyramidal coordination geometry being completed by the pyridine N atom of an *N*,*N*-diethyl­nicotinamide (DENA) ligand. The dihedral angle between the benzene ring and the carboxyl­ate group is 7.06 (11)° in one of the independent DEAB ligands and 4.42 (9)° in the other. The benzene rings of the two independent DEAB ligands are oriented at a dihedral angle of 86.35 (8)°. The pyridine ring is oriented at dihedral angles of 31.43 (6) and 57.92 (7)° with respect to the two benzene rings. In the crystal, weak inter­molecular C—H⋯O inter­actions link the mol­ecules into a three-dimensional network. Weak C—H⋯π inter­actions are also present in the crystal structure.

## Related literature

For niacin, see: Krishnamachari (1974 ▶). For *N*,*N*-diethyl­nicotinamide, see: Bigoli *et al.* (1972 ▶). For related structures, see: Speier & Fulop (1989 ▶); Usubaliev *et al.* (1980 ▶); Hökelek *et al.* (1995 ▶, 2009*a* ▶,*b* ▶,*c* ▶); Necefoğlu *et al.* (2010*a* ▶,*b* ▶).
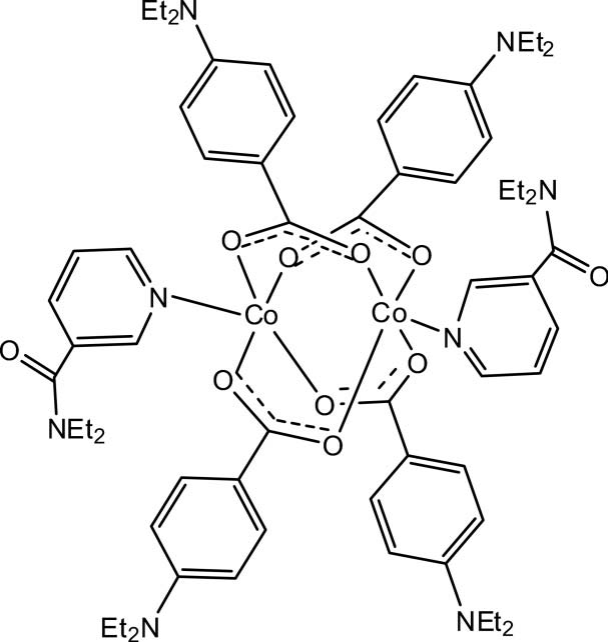


## Experimental

### 

#### Crystal data

[Co_2_(C_11_H_14_NO_2_)_4_(C_10_H_14_N_2_O)_2_]
*M* *_r_* = 1243.25
Monoclinic, 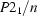

*a* = 10.3518 (2) Å
*b* = 13.4393 (2) Å
*c* = 22.5105 (3) Å
β = 94.189 (2)°
*V* = 3123.32 (9) Å^3^
*Z* = 2
Mo *K*α radiation
μ = 0.60 mm^−1^
*T* = 100 K
0.44 × 0.36 × 0.21 mm

#### Data collection

Bruker Kappa APEXII CCD area-detector diffractometer
Absorption correction: multi-scan (*SADABS*; Bruker, 2005 ▶) *T*_min_ = 0.771, *T*_max_ = 0.881
29642 measured reflections
7775 independent reflections
6209 reflections with *I* > 2σ(*I*)
*R*_int_ = 0.030

#### Refinement

*R*[*F* ^2^ > 2σ(*F* ^2^)] = 0.045
*wR*(*F* ^2^) = 0.119
*S* = 1.04
7775 reflections
385 parameters
1 restraint
H-atom parameters constrained
Δρ_max_ = 1.86 e Å^−3^
Δρ_min_ = −0.63 e Å^−3^

### 

Data collection: *APEX2* (Bruker, 2007 ▶); cell refinement: *SAINT* (Bruker, 2007 ▶); data reduction: *SAINT*; program(s) used to solve structure: *SHELXS97* (Sheldrick, 2008 ▶); program(s) used to refine structure: *SHELXL97* (Sheldrick, 2008 ▶); molecular graphics: *ORTEP-3 for Windows* (Farrugia, 1997 ▶); software used to prepare material for publication: *WinGX* (Farrugia, 1999 ▶) and *PLATON* (Spek, 2009 ▶).

## Supplementary Material

Crystal structure: contains datablocks I, global. DOI: 10.1107/S160053681005004X/xu5109sup1.cif

Structure factors: contains datablocks I. DOI: 10.1107/S160053681005004X/xu5109Isup2.hkl

Additional supplementary materials:  crystallographic information; 3D view; checkCIF report

## Figures and Tables

**Table 1:** Selected bond lengths (Å)

Co1—O1	2.0287 (15)
Co1—O2	2.0262 (16)
Co1—O3	2.0347 (15)
Co1—O4	2.0223 (15)
Co1—N1	2.0702 (18)

**Table 2:** Hydrogen-bond geometry (Å, °) *Cg*1 is the centroid of the C2–C7 ring.

*D*—H⋯*A*	*D*—H	H⋯*A*	*D*⋯*A*	*D*—H⋯*A*
C10—H10*B*⋯O5^i^	0.97	2.49	3.380 (3)	153
C24—H24⋯O5^ii^	0.93	2.57	3.307 (3)	136
C19—H19*A*⋯*Cg*1^iii^	0.97	2.94	3.872 (3)	162
C31—H31*B*⋯*Cg*1^iv^	0.97	2.88	3.637 (2)	136

Symmetry codes: (i) 
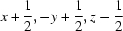
; (ii) 
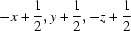
; (iii) 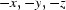; (iv) 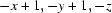.

**Fig. 1.**
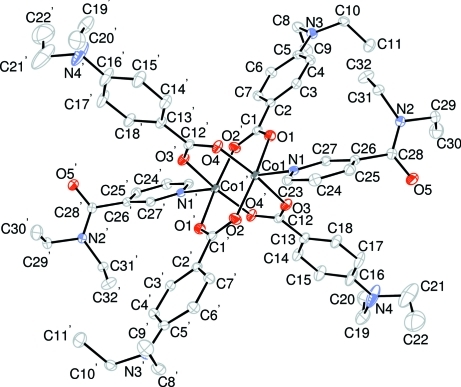
The molecular structure of the title molecule with the atom-numbering scheme. Displacement ellipsoids are drawn at the 50% probability level. The hydrogen atoms are omitted for clarity. Primed atoms are generated by the symmetry operator:(') - x, 1 - y, - z.

## supplementary crystallographic information

### Comment

As a part of our ongoing investigation on transition metal complexes of nicotinamide (NA), one form of niacin (Krishnamachari, 1974), and/or the nicotinic acid derivative *N*,*N*-diethylnicotinamide (DENA), an important respiratory stimulant (Bigoli *et al.*, 1972), the title compound was synthesized and its crystal structure is reported herein.

The title compound is a binuclear compound, consisting of two DENA and four diethylaminobenzoate (DEAB) ligands. The structures of similar complexes of the Cu^2+^ and Zn^2+^ ions, [Cu(C_6_H_5_COO)_2_(C_5_H_5_N)]_2_ (Usubaliev *et al.*, 1980); [Cu(C_6_H_5_CO_2_)_2_(Py)]_2_ (Speier & Fulop, 1989); [Cu_2_(C_6_H_5_COO)_4_(C_10_H_14_N_2_O)_2_] (Hökelek *et al.*, 1995) [Cu_2_(C_8_H_7_O_2_)_4_(C_6_H_6_N_2_O)_2_] (Necefoğlu *et al.*, 2010*a*) [Zn_2_(C_11_H_14_NO_2_)_4_(C_10_H_14_N_2_O)_2_] (Hökelek *et al.*, 2009*a*); [Zn_2_(C_8_H_8_NO_2_)_4_(C_10_H_14_N_2_O)_2_].2H_2_O (Hökelek *et al.*, 2009*b*); [Zn_2_(C_9_H_10_NO_2_)_4_(C_10_H_14_N_2_O)_2_] (Hökelek *et al.*, 2009*c*); [Zn_2_(C_8_H_7_O_2_)_4_(C_10_H_14_N_2_O)_2_] (Necefoğlu *et al.*, 2010*b*) have also been determined. In these structures, the benzoate ion acts as a bidentate ligand.

The title dimeric complex, [Co_2_(DEAB)_4_(DENA)_2_], has a centre of symmetry and two Co^II^ atoms are surrounded by four DEAB groups and two DENA ligands. The DENA ligands are coordinated to Co atoms through pyridine N atoms only. The DEAB groups act as bridging ligands. The Co···Co' distance is 2.6199 (5) Å. The average Co-O distance is 2.0280 (15) Å (Table 1), and four O atoms of the bridging DEAB ligands around each Co atom form a distorted square plane. The Co atom lies 0.1857 (3) Å below the least-squares plane. The average O-Co-O bond angle is 89.36 (7)°. A distorted square-pyramidal arrangement around each Co atom is completed by the pyridine N atom of DENA ligand at 2.0702 (18) Å from the Co atom (Table 1). The N1-Co1···Co1' angle is 171.59 (5)° and the dihedral angle between plane through Co1, O1, O2, C1, Co1', O1', O2', C1' and the plane through Co1, O3, O4, C12, Co1', O3', O4', C12' is 89.51 (6)°. The dihedral angles between the planar carboxylate groups and the adjacent benzene rings A (C2-C7) and B (C13-C18) are 7.06 (11) and 4.42 (9) °, respectively, while that between rings A and B is A/B = 86.35 (8)°. Ring C (N1/C23-C27) is oriented with respect to rings A and B at dihedral angles A/C = 31.43 (6) and B/C = 57.92 (7) °.

In the crystal structure, weak intermolecular C-H···O interactions (Table 2) link the molecules into a two-dimensional network, in which they may be effective in the stabilization of the structure. Two weak C-H···π interactions (Table 2) are also found.

### Experimental

The title compound was prepared by the reaction of CoSO_4_.7H_2_O (1.41 g, 5 mmol) in H_2_O (50 ml) and DENA (1.78 g, 10 mmol) in H_2_O (50 ml) with sodium p-diethylaminobenzoate (2.16 g, 10 mmol) in H_2_O (100 ml). The mixture was filtered and set aside to crystallize at ambient temperature for one week, giving blue single crystals.

### Refinement

H atoms were positioned geometrically with C-H = 0.93, 0.97 and 0.96 Å, for aromatic, methylene and methyl H atoms, respectively, and constrained to ride on their parent atoms, with *U*_iso_(H) = *xU*_eq_(C), where *x* = 1.5 for methyl H and *x* = 1.2 for aromatic H atoms.

### Crystal data

**Table:**

[Co_2_(C_11_H_14_NO_2_)_4_(C_10_H_14_N_2_O)_2_]	*F*(000) = 1316
*M**_r_* = 1243.25	*D*_x_ = 1.322 Mg m^−^^3^
Monoclinic, *P*2_1_/*n*	Mo *K*α radiation, λ = 0.71073 Å
Hall symbol: -P 2yn	Cell parameters from 9913 reflections
*a* = 10.3518 (2) Å	θ = 2.2–28.3°
*b* = 13.4393 (2) Å	µ = 0.60 mm^−^^1^
*c* = 22.5105 (3) Å	*T* = 100 K
β = 94.189 (2)°	Block, blue
*V* = 3123.32 (9) Å^3^	0.44 × 0.36 × 0.21 mm
*Z* = 2	

### Data collection

**Table:**

Bruker Kappa APEXII CCD area-detector diffractometer	7775 independent reflections
Radiation source: fine-focus sealed tube	6209 reflections with *I* > 2σ(*I*)
graphite	*R*_int_ = 0.030
φ and ω scans	θ_max_ = 28.4°, θ_min_ = 1.8°
Absorption correction: multi-scan (*SADABS*; Bruker, 2005)	*h* = −13→11
*T*_min_ = 0.771, *T*_max_ = 0.881	*k* = −17→15
29642 measured reflections	*l* = −28→30

### Refinement

**Table:**

Refinement on *F*^2^	Primary atom site location: structure-invariant direct methods
Least-squares matrix: full	Secondary atom site location: difference Fourier map
*R*[*F*^2^ > 2σ(*F*^2^)] = 0.045	Hydrogen site location: inferred from neighbouring sites
*wR*(*F*^2^) = 0.119	H-atom parameters constrained
*S* = 1.04	*w* = 1/[σ^2^(*F*_o_^2^) + (0.0516*P*)^2^ + 3.5237*P*] where *P* = (*F*_o_^2^ + 2*F*_c_^2^)/3
7775 reflections	(Δ/σ)_max_ < 0.001
385 parameters	Δρ_max_ = 1.86 e Å^−^^3^
1 restraint	Δρ_min_ = −0.63 e Å^−^^3^

### Special details

**Table:**

Geometry. All esds (except the esd in the dihedral angle between two l.s. planes) are estimated using the full covariance matrix. The cell esds are taken into account individually in the estimation of esds in distances, angles and torsion angles; correlations between esds in cell parameters are only used when they are defined by crystal symmetry. An approximate (isotropic) treatment of cell esds is used for estimating esds involving l.s. planes.
Refinement. Refinement of F^2^ against ALL reflections. The weighted R-factor wR and goodness of fit S are based on F^2^, conventional R-factors R are based on F, with F set to zero for negative F^2^. The threshold expression of F^2^ > 2sigma(F^2^) is used only for calculating R-factors(gt) etc. and is not relevant to the choice of reflections for refinement. R-factors based on F^2^ are statistically about twice as large as those based on F, and R- factors based on ALL data will be even larger.

### Fractional atomic coordinates and isotropic or equivalent isotropic displacement parameters (Å^2^)

**Table:**

	*x*	*y*	*z*	*U*_iso_*/*U*_eq_	
Co1	0.08277 (3)	0.50124 (2)	0.046881 (12)	0.01343 (9)	
O1	0.21346 (15)	0.45553 (12)	−0.01024 (7)	0.0193 (3)	
O2	−0.07212 (15)	0.54348 (12)	0.09087 (7)	0.0213 (3)	
O3	0.02198 (15)	0.35760 (11)	0.05110 (7)	0.0204 (3)	
O4	0.12056 (16)	0.64540 (11)	0.02910 (7)	0.0207 (3)	
O5	0.39349 (16)	0.22715 (12)	0.22409 (8)	0.0272 (4)	
N1	0.19986 (17)	0.48455 (13)	0.12480 (8)	0.0155 (4)	
N2	0.54359 (17)	0.28424 (13)	0.16426 (9)	0.0184 (4)	
N3	0.57041 (18)	0.27458 (14)	−0.20266 (8)	0.0192 (4)	
N4	−0.1917 (3)	−0.08369 (19)	0.07254 (13)	0.0586 (9)	
C1	0.1838 (2)	0.44190 (15)	−0.06537 (10)	0.0164 (4)	
C2	0.2870 (2)	0.40483 (15)	−0.10227 (10)	0.0161 (4)	
C3	0.4103 (2)	0.38332 (16)	−0.07660 (10)	0.0174 (4)	
H3	0.4295	0.3974	−0.0365	0.021*	
C4	0.5050 (2)	0.34162 (16)	−0.10906 (10)	0.0181 (4)	
H4	0.5861	0.3275	−0.0904	0.022*	
C5	0.4795 (2)	0.32034 (16)	−0.17039 (10)	0.0169 (4)	
C6	0.3572 (2)	0.34872 (17)	−0.19684 (10)	0.0201 (4)	
H6	0.3395	0.3403	−0.2376	0.024*	
C7	0.2632 (2)	0.38885 (16)	−0.16323 (10)	0.0188 (4)	
H7	0.1828	0.4054	−0.1817	0.023*	
C8	0.5472 (2)	0.25945 (19)	−0.26694 (10)	0.0238 (5)	
H8A	0.6303	0.2546	−0.2840	0.029*	
H8B	0.5035	0.3179	−0.2839	0.029*	
C9	0.4673 (3)	0.1681 (2)	−0.28582 (12)	0.0304 (6)	
H9A	0.4543	0.1663	−0.3285	0.046*	
H9B	0.3849	0.1713	−0.2689	0.046*	
H9C	0.5124	0.1092	−0.2720	0.046*	
C10	0.6810 (2)	0.22247 (17)	−0.17283 (10)	0.0202 (4)	
H10A	0.7188	0.2642	−0.1409	0.024*	
H10B	0.7461	0.2124	−0.2011	0.024*	
C11	0.6460 (3)	0.12205 (18)	−0.14698 (11)	0.0273 (5)	
H11A	0.7227	0.0911	−0.1289	0.041*	
H11B	0.6087	0.0802	−0.1782	0.041*	
H11C	0.5846	0.1316	−0.1175	0.041*	
C12	−0.0614 (2)	0.31422 (16)	0.01624 (10)	0.0173 (4)	
C13	−0.0922 (2)	0.20945 (16)	0.02986 (10)	0.0187 (4)	
C14	−0.1889 (2)	0.15828 (17)	−0.00377 (11)	0.0241 (5)	
H14	−0.2324	0.1899	−0.0361	0.029*	
C15	−0.2217 (3)	0.06189 (18)	0.00965 (12)	0.0313 (6)	
H15	−0.2872	0.0299	−0.0135	0.038*	
C16	−0.1573 (3)	0.01105 (19)	0.05801 (13)	0.0363 (7)	
C17	−0.0571 (3)	0.06262 (18)	0.09112 (12)	0.0322 (6)	
H17	−0.0107	0.0308	0.1225	0.039*	
C18	−0.0274 (2)	0.15955 (17)	0.07739 (11)	0.0237 (5)	
H18	0.0374	0.1924	0.1004	0.028*	
C19	−0.3105 (3)	−0.1314 (2)	0.04357 (14)	0.0422 (7)	
H19A	−0.3446	−0.1795	0.0705	0.051*	
H19B	−0.3764	−0.0813	0.0344	0.051*	
C20	−0.2775 (3)	−0.1820 (2)	−0.01213 (16)	0.0477 (8)	
H20A	−0.3547	−0.2089	−0.0322	0.072*	
H20B	−0.2171	−0.2348	−0.0025	0.072*	
H20C	−0.2393	−0.1348	−0.0377	0.072*	
C21	−0.1063 (5)	−0.1455 (3)	0.11570 (18)	0.0771 (14)	
H21A	−0.1127	−0.2154	0.1052	0.093*	
H21B	−0.0165	−0.1250	0.1149	0.093*	
C22	−0.1526 (4)	−0.1287 (3)	0.1752 (2)	0.0805 (13)	
H22A	−0.1052	−0.1704	0.2037	0.121*	
H22B	−0.2432	−0.1445	0.1745	0.121*	
H22C	−0.1398	−0.0602	0.1863	0.121*	
C23	0.1870 (2)	0.54421 (16)	0.17171 (10)	0.0172 (4)	
H23	0.1288	0.5969	0.1677	0.021*	
C24	0.2571 (2)	0.53051 (17)	0.22588 (10)	0.0202 (4)	
H24	0.2475	0.5741	0.2573	0.024*	
C25	0.3415 (2)	0.45098 (17)	0.23237 (10)	0.0196 (4)	
H25	0.3887	0.4398	0.2685	0.024*	
C26	0.3553 (2)	0.38771 (16)	0.18422 (10)	0.0169 (4)	
C27	0.2831 (2)	0.40772 (16)	0.13115 (10)	0.0165 (4)	
H27	0.2927	0.3663	0.0986	0.020*	
C28	0.4338 (2)	0.29336 (16)	0.19214 (10)	0.0181 (4)	
C29	0.6145 (2)	0.18977 (17)	0.17123 (11)	0.0241 (5)	
H29A	0.6180	0.1699	0.2127	0.029*	
H29B	0.7027	0.2000	0.1607	0.029*	
C30	0.5542 (3)	0.10637 (19)	0.13335 (14)	0.0355 (6)	
H30A	0.6006	0.0457	0.1422	0.053*	
H30B	0.5585	0.1224	0.0920	0.053*	
H30C	0.4653	0.0982	0.1419	0.053*	
C31	0.6028 (2)	0.36507 (17)	0.13149 (10)	0.0209 (5)	
H31A	0.6913	0.3750	0.1478	0.025*	
H31B	0.5555	0.4262	0.1370	0.025*	
C32	0.6032 (3)	0.3432 (2)	0.06586 (12)	0.0313 (6)	
H32A	0.6430	0.3975	0.0463	0.047*	
H32B	0.5157	0.3351	0.0493	0.047*	
H32C	0.6510	0.2833	0.0601	0.047*	

### Atomic displacement parameters (Å^2^)

**Table:**

	*U*^11^	*U*^22^	*U*^33^	*U*^12^	*U*^13^	*U*^23^
Co1	0.01556 (14)	0.01128 (15)	0.01370 (14)	−0.00036 (11)	0.00279 (10)	0.00104 (10)
O1	0.0209 (7)	0.0199 (8)	0.0177 (8)	0.0017 (6)	0.0049 (6)	−0.0005 (6)
O2	0.0182 (7)	0.0254 (8)	0.0207 (8)	0.0029 (7)	0.0043 (6)	−0.0011 (7)
O3	0.0260 (8)	0.0135 (7)	0.0216 (8)	−0.0054 (6)	0.0012 (6)	0.0013 (6)
O4	0.0275 (8)	0.0131 (7)	0.0215 (8)	−0.0015 (6)	0.0008 (6)	0.0037 (6)
O5	0.0278 (9)	0.0215 (9)	0.0339 (10)	0.0041 (7)	0.0120 (7)	0.0105 (7)
N1	0.0158 (8)	0.0128 (8)	0.0185 (9)	−0.0021 (7)	0.0050 (7)	0.0009 (7)
N2	0.0171 (8)	0.0159 (9)	0.0222 (10)	0.0012 (7)	0.0022 (7)	0.0042 (7)
N3	0.0192 (9)	0.0223 (10)	0.0167 (9)	0.0029 (7)	0.0047 (7)	−0.0013 (7)
N4	0.086 (2)	0.0262 (12)	0.0576 (17)	−0.0294 (13)	−0.0359 (15)	0.0207 (12)
C1	0.0200 (10)	0.0109 (9)	0.0190 (10)	−0.0019 (8)	0.0060 (8)	0.0006 (8)
C2	0.0174 (9)	0.0129 (9)	0.0185 (10)	−0.0012 (8)	0.0050 (8)	0.0010 (8)
C3	0.0215 (10)	0.0147 (10)	0.0161 (10)	−0.0001 (8)	0.0025 (8)	0.0008 (8)
C4	0.0172 (10)	0.0180 (10)	0.0193 (10)	0.0011 (8)	0.0020 (8)	0.0000 (8)
C5	0.0174 (10)	0.0156 (10)	0.0185 (10)	−0.0019 (8)	0.0059 (8)	−0.0005 (8)
C6	0.0217 (10)	0.0231 (11)	0.0157 (10)	0.0007 (9)	0.0025 (8)	−0.0026 (8)
C7	0.0165 (10)	0.0195 (11)	0.0204 (11)	0.0008 (8)	0.0017 (8)	−0.0004 (8)
C8	0.0231 (11)	0.0319 (13)	0.0171 (11)	0.0038 (10)	0.0069 (9)	−0.0034 (9)
C9	0.0311 (13)	0.0335 (14)	0.0265 (13)	0.0028 (11)	0.0015 (10)	−0.0082 (11)
C10	0.0183 (10)	0.0204 (11)	0.0226 (11)	0.0016 (9)	0.0068 (8)	−0.0017 (9)
C11	0.0363 (13)	0.0214 (12)	0.0240 (12)	−0.0005 (10)	0.0022 (10)	−0.0002 (9)
C12	0.0200 (10)	0.0134 (10)	0.0190 (10)	−0.0004 (8)	0.0064 (8)	−0.0002 (8)
C13	0.0246 (11)	0.0134 (10)	0.0184 (11)	−0.0022 (8)	0.0026 (8)	0.0006 (8)
C14	0.0320 (12)	0.0181 (11)	0.0212 (11)	−0.0049 (9)	−0.0042 (9)	0.0041 (9)
C15	0.0408 (14)	0.0199 (12)	0.0311 (14)	−0.0116 (11)	−0.0117 (11)	0.0042 (10)
C16	0.0547 (17)	0.0178 (12)	0.0341 (15)	−0.0142 (12)	−0.0137 (13)	0.0082 (10)
C17	0.0472 (15)	0.0181 (12)	0.0289 (13)	−0.0091 (11)	−0.0138 (12)	0.0103 (10)
C18	0.0306 (12)	0.0166 (11)	0.0230 (12)	−0.0071 (9)	−0.0037 (9)	0.0028 (9)
C19	0.0583 (18)	0.0207 (13)	0.0479 (17)	−0.0097 (13)	0.0050 (14)	0.0037 (12)
C20	0.0459 (17)	0.0360 (16)	0.062 (2)	0.0028 (14)	0.0069 (15)	0.0019 (14)
C21	0.113 (3)	0.047 (2)	0.065 (2)	−0.049 (2)	−0.030 (2)	0.0196 (17)
C22	0.070 (2)	0.070 (3)	0.099 (3)	−0.024 (2)	−0.007 (2)	0.019 (2)
C23	0.0184 (10)	0.0137 (10)	0.0200 (11)	−0.0011 (8)	0.0040 (8)	−0.0006 (8)
C24	0.0240 (11)	0.0171 (10)	0.0197 (11)	−0.0024 (9)	0.0030 (9)	−0.0039 (8)
C25	0.0207 (10)	0.0200 (11)	0.0180 (10)	−0.0018 (9)	0.0001 (8)	0.0019 (9)
C26	0.0164 (9)	0.0152 (10)	0.0194 (11)	−0.0009 (8)	0.0036 (8)	0.0030 (8)
C27	0.0175 (10)	0.0145 (10)	0.0178 (10)	−0.0002 (8)	0.0045 (8)	−0.0004 (8)
C28	0.0188 (10)	0.0165 (10)	0.0191 (11)	0.0012 (8)	0.0013 (8)	0.0018 (8)
C29	0.0205 (11)	0.0220 (12)	0.0302 (13)	0.0057 (9)	0.0039 (9)	0.0080 (10)
C30	0.0405 (15)	0.0180 (12)	0.0485 (17)	0.0036 (11)	0.0076 (13)	−0.0009 (11)
C31	0.0177 (10)	0.0175 (11)	0.0278 (12)	−0.0018 (8)	0.0037 (9)	0.0037 (9)
C32	0.0389 (14)	0.0290 (13)	0.0269 (13)	−0.0101 (11)	0.0086 (11)	0.0037 (10)

### Geometric parameters (Å, °)

**Table:**

Co1—Co1^i^	2.6199 (5)	C12—C13	1.481 (3)
Co1—O1	2.0287 (15)	C13—C14	1.392 (3)
Co1—O2	2.0262 (16)	C14—C15	1.378 (3)
Co1—O3	2.0347 (15)	C14—H14	0.9300
Co1—O4	2.0223 (15)	C15—C16	1.411 (4)
Co1—N1	2.0702 (18)	C15—H15	0.9300
O1—C1	1.270 (3)	C17—C16	1.414 (4)
O2—C1^i^	1.268 (3)	C17—H17	0.9300
O3—C12	1.266 (3)	C18—C13	1.393 (3)
O4—C12^i^	1.273 (3)	C18—C17	1.379 (3)
O5—C28	1.236 (3)	C18—H18	0.9300
N1—C23	1.340 (3)	C19—C20	1.488 (4)
N1—C27	1.346 (3)	C19—H19A	0.9700
N2—C28	1.343 (3)	C19—H19B	0.9700
N2—C29	1.469 (3)	C20—H20A	0.9600
N2—C31	1.472 (3)	C20—H20B	0.9600
N3—C8	1.464 (3)	C20—H20C	0.9600
N4—C16	1.368 (3)	C21—N4	1.513 (4)
N4—C19	1.494 (4)	C21—C22	1.474 (6)
C1—O2^i^	1.268 (3)	C21—H21A	0.9700
C1—C2	1.486 (3)	C21—H21B	0.9700
C2—C7	1.393 (3)	C22—H22A	0.9600
C3—C2	1.392 (3)	C22—H22B	0.9600
C3—C4	1.384 (3)	C22—H22C	0.9600
C3—H3	0.9300	C23—C24	1.385 (3)
C4—C5	1.416 (3)	C23—H23	0.9300
C4—H4	0.9300	C24—C25	1.381 (3)
C5—N3	1.375 (3)	C24—H24	0.9300
C5—C6	1.412 (3)	C25—H25	0.9300
C6—C7	1.385 (3)	C26—C25	1.393 (3)
C6—H6	0.9300	C26—C28	1.510 (3)
C7—H7	0.9300	C27—C26	1.388 (3)
C8—C9	1.523 (3)	C27—H27	0.9300
C8—H8A	0.9700	C29—C30	1.515 (4)
C8—H8B	0.9700	C29—H29A	0.9700
C9—H9A	0.9600	C29—H29B	0.9700
C9—H9B	0.9600	C30—H30A	0.9600
C9—H9C	0.9600	C30—H30B	0.9600
C10—N3	1.462 (3)	C30—H30C	0.9600
C10—C11	1.524 (3)	C31—C32	1.506 (3)
C10—H10A	0.9700	C31—H31A	0.9700
C10—H10B	0.9700	C31—H31B	0.9700
C11—H11A	0.9600	C32—H32A	0.9600
C11—H11B	0.9600	C32—H32B	0.9600
C11—H11C	0.9600	C32—H32C	0.9600
C12—O4^i^	1.273 (3)		
			
O1—Co1—Co1^i^	84.99 (5)	C15—C14—C13	121.6 (2)
O1—Co1—O3	88.00 (7)	C15—C14—H14	119.2
O1—Co1—N1	97.35 (7)	C13—C14—H14	119.2
O2—Co1—Co1^i^	84.43 (5)	C14—C15—C16	120.9 (2)
O2—Co1—O1	169.35 (6)	C14—C15—H15	119.5
O2—Co1—O3	89.11 (7)	C16—C15—H15	119.5
O2—Co1—N1	92.95 (7)	N4—C16—C15	121.2 (2)
O3—Co1—Co1^i^	80.75 (5)	N4—C16—C17	121.6 (2)
O3—Co1—N1	91.24 (6)	C15—C16—C17	117.2 (2)
O4—Co1—Co1^i^	88.70 (5)	C16—C17—H17	119.6
O4—Co1—O1	90.98 (7)	C18—C17—C16	120.8 (2)
O4—Co1—O2	89.99 (7)	C18—C17—H17	119.6
O4—Co1—O3	169.45 (6)	C13—C18—H18	119.2
O4—Co1—N1	99.31 (7)	C17—C18—C13	121.6 (2)
N1—Co1—Co1^i^	171.59 (5)	C17—C18—H18	119.2
C1—O1—Co1	122.57 (14)	N4—C19—H19A	109.8
C1^i^—O2—Co1	123.37 (14)	N4—C19—H19B	109.8
C12—O3—Co1	127.39 (14)	C20—C19—N4	109.3 (3)
C12^i^—O4—Co1	118.59 (14)	C20—C19—H19A	109.8
C23—N1—C27	118.36 (19)	C20—C19—H19B	109.8
C23—N1—Co1	121.14 (14)	H19A—C19—H19B	108.3
C27—N1—Co1	120.25 (15)	C19—C20—H20A	109.5
C28—N2—C29	117.52 (18)	C19—C20—H20B	109.5
C28—N2—C31	124.37 (18)	C19—C20—H20C	109.5
C29—N2—C31	117.91 (18)	H20A—C20—H20B	109.5
C5—N3—C10	120.94 (18)	H20A—C20—H20C	109.5
C5—N3—C8	121.00 (18)	H20B—C20—H20C	109.5
C10—N3—C8	117.01 (18)	N4—C21—H21A	110.4
C16—N4—C19	121.0 (2)	N4—C21—H21B	110.4
C16—N4—C21	120.9 (3)	C22—C21—N4	106.6 (4)
C19—N4—C21	117.9 (2)	C22—C21—H21A	110.4
O1—C1—C2	117.53 (19)	C22—C21—H21B	110.4
O2^i^—C1—O1	124.6 (2)	H21A—C21—H21B	108.6
O2^i^—C1—C2	117.88 (19)	C21—C22—H22A	109.5
C3—C2—C1	120.80 (19)	C21—C22—H22B	109.5
C3—C2—C7	117.9 (2)	C21—C22—H22C	109.5
C7—C2—C1	121.32 (19)	H22A—C22—H22B	109.5
C2—C3—H3	119.0	H22A—C22—H22C	109.5
C4—C3—C2	121.9 (2)	H22B—C22—H22C	109.5
C4—C3—H3	119.0	N1—C23—C24	122.6 (2)
C3—C4—C5	120.5 (2)	N1—C23—H23	118.7
C3—C4—H4	119.7	C24—C23—H23	118.7
C5—C4—H4	119.7	C23—C24—H24	120.6
N3—C5—C4	121.31 (19)	C25—C24—C23	118.9 (2)
N3—C5—C6	121.7 (2)	C25—C24—H24	120.6
C6—C5—C4	117.01 (19)	C24—C25—C26	119.3 (2)
C5—C6—H6	119.4	C24—C25—H25	120.3
C7—C6—C5	121.3 (2)	C26—C25—H25	120.3
C7—C6—H6	119.4	C25—C26—C28	120.36 (19)
C2—C7—H7	119.4	C27—C26—C25	118.2 (2)
C6—C7—C2	121.2 (2)	C27—C26—C28	121.0 (2)
C6—C7—H7	119.4	N1—C27—C26	122.7 (2)
N3—C8—C9	115.8 (2)	N1—C27—H27	118.7
N3—C8—H8A	108.3	C26—C27—H27	118.7
N3—C8—H8B	108.3	O5—C28—N2	122.6 (2)
C9—C8—H8A	108.3	O5—C28—C26	118.14 (19)
C9—C8—H8B	108.3	N2—C28—C26	119.22 (19)
H8A—C8—H8B	107.4	N2—C29—C30	113.4 (2)
C8—C9—H9A	109.5	N2—C29—H29A	108.9
C8—C9—H9B	109.5	N2—C29—H29B	108.9
C8—C9—H9C	109.5	C30—C29—H29A	108.9
H9A—C9—H9B	109.5	C30—C29—H29B	108.9
H9A—C9—H9C	109.5	H29A—C29—H29B	107.7
H9B—C9—H9C	109.5	C29—C30—H30A	109.5
N3—C10—C11	113.57 (19)	C29—C30—H30B	109.5
N3—C10—H10A	108.9	C29—C30—H30C	109.5
N3—C10—H10B	108.9	H30A—C30—H30B	109.5
C11—C10—H10A	108.9	H30A—C30—H30C	109.5
C11—C10—H10B	108.9	H30B—C30—H30C	109.5
H10A—C10—H10B	107.7	N2—C31—C32	112.25 (19)
C10—C11—H11A	109.5	N2—C31—H31A	109.2
C10—C11—H11B	109.5	N2—C31—H31B	109.2
C10—C11—H11C	109.5	C32—C31—H31A	109.2
H11A—C11—H11B	109.5	C32—C31—H31B	109.2
H11A—C11—H11C	109.5	H31A—C31—H31B	107.9
H11B—C11—H11C	109.5	C31—C32—H32A	109.5
O3—C12—O4^i^	124.6 (2)	C31—C32—H32B	109.5
O3—C12—C13	117.19 (19)	C31—C32—H32C	109.5
O4^i^—C12—C13	118.26 (19)	H32A—C32—H32B	109.5
C14—C13—C12	121.0 (2)	H32A—C32—H32C	109.5
C14—C13—C18	117.8 (2)	H32B—C32—H32C	109.5
C18—C13—C12	121.2 (2)		
			
Co1^i^—Co1—O1—C1	0.51 (16)	C21—N4—C16—C17	−13.4 (6)
O2—Co1—O1—C1	7.0 (4)	C16—N4—C19—C20	88.1 (4)
O3—Co1—O1—C1	81.40 (16)	C21—N4—C19—C20	−88.3 (4)
O4—Co1—O1—C1	−88.10 (16)	O1—C1—C2—C3	1.8 (3)
N1—Co1—O1—C1	172.39 (16)	O1—C1—C2—C7	−179.3 (2)
Co1^i^—Co1—O2—C1^i^	−2.46 (16)	O2^i^—C1—C2—C3	−177.3 (2)
O1—Co1—O2—C1^i^	−9.0 (5)	O2^i^—C1—C2—C7	1.5 (3)
O3—Co1—O2—C1^i^	−83.25 (17)	C1—C2—C7—C6	−176.1 (2)
O4—Co1—O2—C1^i^	86.23 (17)	C3—C2—C7—C6	2.8 (3)
N1—Co1—O2—C1^i^	−174.45 (17)	C4—C3—C2—C1	175.0 (2)
Co1^i^—Co1—O3—C12	−0.78 (17)	C4—C3—C2—C7	−3.9 (3)
O1—Co1—O3—C12	−86.04 (18)	C2—C3—C4—C5	0.7 (3)
O2—Co1—O3—C12	83.71 (18)	C3—C4—C5—N3	−176.9 (2)
O4—Co1—O3—C12	−1.4 (5)	C3—C4—C5—C6	3.5 (3)
N1—Co1—O3—C12	176.65 (18)	C4—C5—N3—C8	−175.4 (2)
Co1^i^—Co1—O4—C12^i^	−0.42 (16)	C4—C5—N3—C10	16.7 (3)
O1—Co1—O4—C12^i^	84.55 (16)	C6—C5—N3—C8	4.2 (3)
O2—Co1—O4—C12^i^	−84.85 (16)	C6—C5—N3—C10	−163.7 (2)
O3—Co1—O4—C12^i^	0.2 (5)	N3—C5—C6—C7	175.8 (2)
N1—Co1—O4—C12^i^	−177.84 (16)	C4—C5—C6—C7	−4.6 (3)
O1—Co1—N1—C23	148.47 (16)	C5—C6—C7—C2	1.5 (3)
O1—Co1—N1—C27	−37.35 (16)	C11—C10—N3—C5	75.1 (3)
O2—Co1—N1—C23	−34.21 (17)	C11—C10—N3—C8	−93.3 (2)
O2—Co1—N1—C27	139.96 (16)	O3—C12—C13—C14	175.7 (2)
O3—Co1—N1—C23	−123.39 (16)	O3—C12—C13—C18	−3.0 (3)
O3—Co1—N1—C27	50.79 (16)	O4^i^—C12—C13—C14	−3.9 (3)
O4—Co1—N1—C23	56.26 (17)	O4^i^—C12—C13—C18	177.4 (2)
O4—Co1—N1—C27	−129.56 (16)	C12—C13—C14—C15	−177.7 (2)
Co1—O1—C1—O2^i^	1.2 (3)	C18—C13—C14—C15	1.0 (4)
Co1—O1—C1—C2	−177.85 (13)	C13—C14—C15—C16	−0.5 (4)
Co1—O3—C12—O4^i^	1.4 (3)	C14—C15—C16—N4	178.3 (3)
Co1—O3—C12—C13	−178.13 (14)	C14—C15—C16—C17	−1.0 (5)
Co1—N1—C23—C24	175.15 (16)	C18—C17—C16—N4	−177.2 (3)
C27—N1—C23—C24	0.9 (3)	C18—C17—C16—C15	2.0 (5)
Co1—N1—C27—C26	−174.00 (16)	C17—C18—C13—C14	0.0 (4)
C23—N1—C27—C26	0.3 (3)	C17—C18—C13—C12	178.7 (2)
C29—N2—C28—O5	2.0 (3)	C13—C18—C17—C16	−1.6 (4)
C29—N2—C28—C26	−177.65 (19)	C22—C21—N4—C16	92.5 (4)
C31—N2—C28—O5	−172.7 (2)	C22—C21—N4—C19	−91.1 (4)
C31—N2—C28—C26	7.6 (3)	N1—C23—C24—C25	−1.5 (3)
C28—N2—C29—C30	77.2 (3)	C23—C24—C25—C26	0.9 (3)
C31—N2—C29—C30	−107.8 (2)	C27—C26—C25—C24	0.2 (3)
C28—N2—C31—C32	−115.2 (2)	C28—C26—C25—C24	−172.4 (2)
C29—N2—C31—C32	70.2 (3)	C25—C26—C28—O5	66.0 (3)
C5—N3—C8—C9	−83.0 (3)	C25—C26—C28—N2	−114.4 (2)
C10—N3—C8—C9	85.4 (2)	C27—C26—C28—O5	−106.4 (3)
C19—N4—C16—C15	−8.9 (5)	C27—C26—C28—N2	73.2 (3)
C19—N4—C16—C17	170.2 (3)	N1—C27—C26—C25	−0.9 (3)
C21—N4—C16—C15	167.4 (4)	N1—C27—C26—C28	171.70 (19)

Symmetry codes: (i) −*x*, −*y*+1, −*z*.

### Hydrogen-bond geometry (Å, °)

**Table:**

Cg1 is the centroid of the C2–C7 ring.

**Table:**

*D*—H···*A*	*D*—H	H···*A*	*D*···*A*	*D*—H···*A*
C10—H10B···O5^ii^	0.97	2.49	3.380 (3)	153
C24—H24···O5^iii^	0.93	2.57	3.307 (3)	136
C19—H19A···Cg1^iv^	0.97	2.94	3.872 (3)	162
C31—H31B···Cg1^v^	0.97	2.88	3.637 (2)	136

Symmetry codes: (ii) *x*+1/2, −*y*+1/2, *z*−1/2; (iii) −*x*+1/2, *y*+1/2, −*z*+1/2; (iv) −*x*, −*y*, −*z*; (v) −*x*+1, −*y*+1, −*z*.
